# Administration of the Second Dose of mRNA COVID-19 Vaccine to a Woman With Immediate Reaction to the First Dose

**DOI:** 10.7759/cureus.36064

**Published:** 2023-03-13

**Authors:** Evaggelia Apostolidou, Konstantina Dimitriou, Anastasia Papadopoulou, Nikolaos Mikos, Evangelia Kompoti

**Affiliations:** 1 Allergology, General Hospital of Athens Laiko, Athens, GRC

**Keywords:** anaphylatoxins, complement-mediated, life threatening anaphylaxis, allergic reaction, covid-19 vaccine

## Abstract

Vaccines constitute the most effective public health intervention as they prevent the spread of infectious diseases and reduce disease severity and mortality. Allergic reactions can occur during vaccination. Systemic anaphylaxis is a severe, life-threatening allergic reaction which can rarely occur after vaccination. There is limited data suggesting that the majority of the patients with immediate and potentially allergic reactions after the first dose of coronavirus disease 2019 (COVID-19) can receive the second dose. A 39-year-old woman was admitted to our department after presenting anaphylactic reaction following the first dose of mRNA COVID-19 vaccine (BNT162b2). A few days later, she contacted our department and was admitted for an allergy work-up on mRNA COVID-19 vaccine and its compound polyethylene glycol (PEG). Thereafter, she completed the vaccination procedure having received pretreatment under our guidance. Confirmed allergic reactions to vaccines are customarily attributed to the inactive ingredients, or excipients like PEG and polysorbate. The latest are used to improve water-solubility in vaccines. PEG itself has not been previously used in a vaccine but polysorbate has been identified as a rare cause of allergic reactions to vaccines. It has been reported that the interaction of the immune system with lipidic nanoparticle therapeutics could result in hypersensitivity reactions (HSRs), referred to as complement activation related pseudoallergy (CARPA), which is classified as non-IgE-mediated pseudoallergy caused by the activation of the complement system.

## Introduction

Vaccination is extremely effective for public health. Vaccines not only control, but they also prevent the expansion of infectious diseases and reduce mortality. As with every other medication, allergic reactions can take place during vaccination. System anaphylaxis is a severe, life-threatening allergic reaction which can rarely occur after vaccination. Allergic reactions following mRNA COVID-19 vaccination account to be as high as 2%, with anaphylaxis presenting in up to 2.5 per 10.000 individuals [1]. However, most of the referred allergic reactions are mild or self-limited. There is limited data suggesting that the majority of the patients with immediate and potentially allergic reactions after the first dose of COVID-19 can receive the second dose. We report a case of a woman who experienced an anaphylactic reaction after the first dose of mRNA vaccine (BNT162b2) but she completed the two-shots vaccination protocol procedure having received pretreatment under our guidance. Very few cases like the aforementioned one have been reported so far.

## Case presentation

A 39-year-old woman was admitted to our department for allergy investigation after developing an anaphylactic reaction following the first dose of mRNA COVID-19 vaccine. She complained of pruritus and redness in her chest, 25 min after the vaccine shot, with gradual spreading in the eyes and ears, particularly the left one which was slightly swollen. After a few minutes, she mentioned a burning throat feeling, nose congestion, and catarrh. Finally, she reported dyspnea and slight hoarseness. On physical examination, the patient was conscious and oriented, afebrile, blood pressure: 75/55 mmHg in right arm, pulse: 119 beats per minute, SpO2: 89% on room air. On respiratory system evaluation, bilateral wheezing was present. She was treated with epinephrine intramuscularly, dimethindene, methylprednisolone 125 mg intravenously, and salbutamol/budesonide with a nebulizer by the doctor of the vaccination center. She received O2 by a nasal cannula and recovered immediately. Three days after the vaccine, she reported pruritus at the chest, back, tinnitus, and swelling in the tongue. She received levocetirizine 5 mg and two doses of prednisolone 5 mg, 3 h after the beginning of the symptoms, under the guidance of her general physician. The symptoms lasted 2 h and she fully recovered after 5 h.

A few days later, she contacted our department and was admitted for an allergy work-up on mRNA COVID-19 vaccine (BNT162b2) and its compound polyethylene glycol (PEG). Both were examined via skin prick test and intradermal test in the appropriate dilutions (1/1000, 1/100, 1/10) which resulted negative for immediate type reactions, except for 1/10 of mRNA vaccine which came positive 3 h later. Therefore, it did not show evidence of IgE sensitization to the above substances. However, the prognostic value for these tests has not been validated yet. We performed a laboratory evaluation which revealed tryptase levels: 4.17 ng/mL (reference value: <11.50 ng/mL). The patient had a history of allergic reaction to moxifloxacin (acute urticaria on the second day of IV administration for treating corpus luteum rupture) and free further medical history. She wished to complete the vaccination procedure against COVID-19 due to professional reasons.

She received a pretreatment with rupatadine 10 mg (two tablets per day) and montelukast 10 mg (one tablet per day) for 3 days before the second dose’s appointment and on the day of the second dose administration, an i.v. cannula was set. Thirty minutes later, she received the vaccination shot. She remained in the allergology department under close monitoring for 4 h and did not present any symptoms or abnormalities in her vital signs, so she was discharged safely.

## Discussion

Allergic reactions after vaccination including severe anaphylaxis are mainly IgE mediated. They usually take place within 30 min after the vaccine shot. They often present with generalized pruritus, erythema, urticarial rashes, coughing, wheezing, hypotension, and angioedema. The tolerance of a second vaccination dose either doubts on the allergic nature of the reaction being reported after the initial dose, or supports an allergic, but non-IgE-mediated mechanism in which symptoms can typically be avoided with premedications [2]. For example, IgG-mediated and complement-mediated reactions which resemble anaphylaxis symptoms have also been described. Clinical symptoms of these non-IgE-mediated reactions include hypo/hypertension and dyspnea (Table 1). The rate of severe anaphylactic reactions to vaccines has been assessed at 1.31 (95% CI, 0.90-1.84) per million vaccine doses, so they account as rare [3].

**Table 1:** Symptoms of pseudoallergy.

Cardiovascular	Broncho-pulmonary	Hematological	Mucocutaneous	Gastro-intestinal	Neuro-somatic	Systemic
Angioedema	Apnea	Granulopenia	Cyanosis	Bloating	Back pain	Chills
Arrhythmia	Bronchospasm	Leucopenia	Erythema	Cramping	Chest pain	Diaphoresis
Cardiogenic shock	Coughing	Lymphopenia	Flushing	Diarrhea	Chest tightness	Feeling of warmth
Edema	Dyspnea	Rebound leukocytosis	Nasal congestion	Metallic taste	Confusion	Fever
Hypertension	Hoarseness	Rebound granulocytosis	Rash	Nausea	Dizziness	Loss of consciousness
Hypotension	Laryngospasm	Thrombocytopenia	Rhinitis	Vomiting	Feeling of imminent death	Rigors
Hypoxia	Respiratory distress		Swelling		Fright	Sweating
Myocardial infarction	Shortness of breath		Tearing		Headache	Wheezing
Tachycardia	Sneezing		Urticaria		Panic	
Ventricular fibrillation	Stridor					
Syncope						

Confirmed allergic reactions following vaccination are rarely attributed to the active but commonly to the inactive components, or excipients, including egg protein, formaldehyde, gelatin, neomycin, or thimerosal [4]. Other adjuvants, like PEG and polysorbate, are used to upgrade water-solubility in drugs and vaccines. Polysorbate has been identified as a rare cause of allergic reactions to vaccines, however, PEG itself has not previously been used in a vaccine. First dose reactions to vaccines containing polysorbate may have occurred due to prior sensitization from polysorbate80 [5]. The designation of PEG, as a bioinert, is a thermoelastic linear hydrophilic polymer with the molecular formula (C2nH4n+2On+1). It is a non-toxic and non-ionic etherdiol with a GRAS (generally recognized as safe) determination and an increasing utility as a food additive and an additive in pharmaceutical industry. PEG has been used for surface modification of nanocarriers such as liposomes, nanoparticles and therapeutic proteins, with the purpose to increase their half-life circulation [6].

It seems that the immune system’s interaction with lipidic nanoparticle therapeutics could end up in hypersensitivity reactions (HSRs), which are known as complement activation related pseudoallergy (CARPA). The latter is caused by the activation of the complement and finally categorized as non-IgE-mediated reaction [7, 8-12]. Such HSRs may occur straight away when patients exposed to lipid excipients, including lipid nanoparticles, for the first time, without prior sensitization. As for the symptoms, they usually decline or vanish on subsequent treatment. Consequently, such immunological responses are named “pseudoallergy” [10-12]. Around 77% of adverse drug effects are non-IgE mediated (~340,000/year) [13].

As the main responsibility for the body’s immune regulation, the complement system has major role for the defense against foreign intruders. As early as the 1970s, it is reported that the agent for radiocontrast media’s HSRs was the complement activation [14-15]. Hugli [16] described complement activation and depicted the structure and functions of anaphylatoxins, mainly C3a and C5a, in adverse drug reactions mediated by the complement system. Later, Szebeni by discovering receptor-mediated mast cell activation, set CARPA an independent class of type I reactions [7].

The precise mechanism of liposome-induced CARPA is not yet illustrated. It possibly includes many different cellular (secretory, blood, and effector cells) and molecular procedures (anaphylatoxins, allergomedins). Anaphylatoxins such as C5a and C3a are released, soon after complement activation, and with their turn anaphylatoxins trigger macrophages, basophils, and mast cells through their specific receptors to be activated. Then, these cells secrete a variety of vasoactive inflammatory mediators, referred to as allergomedins, involving tryptase, histamine, platelet activating factor (PAF), and leukotrienes (LTB2, LTD4, LTC4, PGD2, TXA2, and LTE4) [7, 17]. The released mediators bind to their receptors on autonomic effector cells such as endothelial cell and smooth muscle cells, causing their activation, resulting in CARPA [18]. Another possible mechanism for liposome-induced CARPA is the ‘double hit hypothesis’ [7]. It is suggested that CARPA is controlled by two strikes on immune cells (basophils, mast cells, tissue macrophages). The first hit is the signal of anaphylatoxins , and the second one is the attachment of the former immune cells, via surface receptors, with molecules or reaction-trigger drugs. The surface receptors are linked to the signal transduction network, which triggers secretory paths. The anaphylatoxins induce secretion of vasoactive secondary mediators via binding to their respective receptors (anaphylatoxins receptors, ATR) on mast cells or pulmonary intravascular macrophages. In the same way, direct binding of the PEG coupled to liposome surfaces to these cells occurs through surface receptors or pattern recognition receptors (PRRs), toll-like receptors (TLR2 and TLR4) or other danger signaling receptors (DSRs) on mast cells and macrophages that might elicit a secretory response [6].

There are approaches to minimize the risk of clinical reaction, with premedication, desensitization, and inhibition of C activation. Pretreatment with anti-allergic drugs such as antihistamines, montelukast and, in some cases, corticosteroids can moderate the gravity of CARPA, setting this approach as the currently standard clinical process [14, 19].

## Conclusions

Although allergic reaction to a vaccine constitutes a relevant contraindication for a second dose, it is feasible after the appropriate premedication, predominantly in cases of pseudoallergy, where CARPA is involved.
